# Chinese Herbal Medicine as Adjunctive Therapy to Chemotherapy for Breast Cancer: A Systematic Review and Meta-Analysis

**DOI:** 10.1155/2016/3281968

**Published:** 2016-04-10

**Authors:** Xu Sun, Xing Zhang, Jia-Yun Nian, Jiao Guo, Yi Yin, Gan-Lin Zhang, Ming-Wei Yu, Yi Zhang, Xiao-Min Wang, Guo-Wang Yang, Lin Yang, Pei-Yu Cheng, Jin-Ping Li

**Affiliations:** ^1^Department of Oncology, Beijing Hospital of Traditional Chinese Medicine, Capital Medical University, Beijing 100010, China; ^2^Beijing University of Chinese Medicine, Beijing 100029, China; ^3^Department of Oncology, Third Affiliated Hospital of Henan College of Traditional Chinese Medicine, Zhengzhou 450000, China; ^4^Department of Medical Biochemistry and Microbiology, Uppsala University, 75123 Uppsala, Sweden

## Abstract

Chinese herbal medicine (CHM) has been increasingly employed during therapy for breast cancer, but its efficacy remains a matter of debate. This systematic review examined randomized controlled trials to provide a critical evaluation of this treatment. The results demonstrated that the combined use of CHM with chemotherapy may improve the immediate tumor response and reduce chemotherapy-associated adverse events. Our findings highlight the poor quality of Chinese studies, and additional well-designed randomized controlled trials addressing the role of CHM are warranted. The lack of molecular-based evidence for CHM and Zheng has resulted in a limited understanding and acceptance of CHM and traditional Chinese medicine in Western countries. We believe that researchers should immediately explore a CHM-based cure, and CHM should be applied to routine care as soon as conclusive data are available.

## 1. Introduction

Breast cancer is the leading cause of cancer-related deaths in women worldwide [1]. Breast cancer has been one of the fastest growing cancers in China over the past 30 years, with an incidence approaching approximately 96%, which is only slightly lower than that of lung cancer [2].

Chemotherapy plays a key role in the systemic treatment of breast cancer, and it is the cornerstone of therapy for patients who are not candidates for endocrine therapy [3]. Adjuvant chemotherapy can increase the pathological complete response (CR) rate and improve survival in most patients with early stage breast cancer [4–6]. The primary objectives of treatment are palliation and improved survival for the vast majority of metastatic breast cancers, which are incurable.

The chemical agents used in chemotherapy are selectively destructive to malignant tissues, but these agents also damage healthy tissue, which results in adverse side effects that negatively impact compliance with cancer treatment. Therefore, there is a clinical need to find an intervention to manage the adverse side effects of chemotherapy and increase patient tolerance and well-being.

Many breast cancer patients take complementary and alternative medicine (CAM), usually in combination with anticancer treatments, and the global use of CAM continues to increase dramatically [7–9]. The application of CAM in Western countries ranges from 9% to 69% [7, 8, 10–15]. Notably, these studies highlighted the use of traditional Chinese herbal medicine (CHM) for breast cancer.

Traditional Chinese medicine (TCM) was developed thousands of years ago, long before the advent of modern science. The use of TCM-based CHM for breast cancer has been described in Chinese medical texts for more than 2000 years [16, 17]. CHM, including botanical, animal, and mineral agents, used by TCM physicians aims to control the side effects and toxicities of cancer therapies, which improves the patient's quality of life (QoL), prevents recurrence, and prolongs survival [18].

Most studies on the clinical efficacy of CHM are based on either personal experience or case reports. Therefore, it is difficult to reach evidence-based conclusions. We conducted this systematic review to evaluate the efficacy of CHM as an adjunctive therapy to chemotherapy for the treatment of breast cancer compared with the use of chemotherapy alone because of the high prevalence of breast cancer in women and the frequent use of chemotherapy in women who present to TCM practitioners.

## 2. Methods

This systematic review was conducted using the PRISMA statement [19]. We developed a protocol prior to conducting the review.

### 2.1. Databases

Two authors independently searched for publications dated as late as August 30, 2014, in the following electronic databases: MEDLINE via OvidSP, EMBASE via OvidSP (as shown in the list below), CINAHL via OvidSP, AMED via OvidSP, PubMed, CENTRAL via OvidSP, Chinese BioMedical Literature Database (CBM), the Chinese database CNKI, Wan Fang, and VIP. The following terms were used in the search: breast cancer, breast neoplasm, breast tumor, mammary cancer, mammary neoplasm, human mammary carcinomas, chemotherapy, Chinese herbals, Chinese herbal drugs, Chinese medicine, traditional Chinese medicine, CHM, TCM, herbals, Kampo, herbal therapy, complementary medicine, alternative medicine, plants, and botany. A manual review of the reference lists of all accepted papers was also conducted.

*Search Strategy for EMBASE via OvidSP to Identify Potential Articles*

(1) Clinical trial/
(2) Randomized controlled trial/
(3) Randomization/
(4) Single blind procedure/
(5) Double blind procedure/
(6) Crossover procedure/
(7) Placebo/
(8) Randomized controlled trial$.tw.
(9) Rct.tw.
(10) Random allocation.tw.
(11) Randomly allocated.tw.
(12) Allocated randomly.tw.
(13) (allocated adj2 random).tw.
(14) Single blind$.tw.
(15) Double blind$.tw.
(16) ((treble or triple) adj (blind$)).tw.
(17) Placebo$.tw.
(18) Prospective study/
(19) Or/1–18
(20) Case study/
(21) Case report.tw.
(22) Abstract report/or letter/
(23) Or/20–22
(24) 19 not 23
(25) exp breast neoplasms/
(26) (breast adj5 neoplasm$).ti,ab.
(27) (breast adj5 cancer$).ti,ab.
(28) (breast adj5 tumo$).ti,ab.
(29) (breast adj5 carcinoma$).ti,ab.
(30) (breast adj5 adenocarcinoma$).ti,ab.
(31) (breast adj5 dcis).ti,ab.
(32) (breast adj5 ductsl).ti,ab.
(33) (breast adj5 sarcoma$).ti,ab.
(34) (breast adj5 infiltrating).ti,ab.
(35) (breast adj5 intraductal).ti,ab.
(36) (breast adj5 lobular).ti,ab.
(37) (breast adj5 medullary).ti,ab.
(38) Or/25–37
(39) exp chinese medicine/
(40) exp medicine, east asian traditional/or exp medicine, chinese traditional/or exp medicine, kampo/or exp medicine, korean traditional/or exp medicine, mongolian traditional/
(41) exp complementary medicine/
(42) exp traditional chinese medicine/
(43) Or/39–42
(44) 38 and 43
(45) 24 and 44

### 2.2. Inclusion Criteria

The inclusion criteria were as follows. (1)* Types of Studies*. There were randomized controlled trials (RCTs) with two arms, without blinding or language restrictions. (2)* Types of Participants*. They were female breast cancer patients who were diagnosed using pathological sections and treated with chemotherapy. (3)* Types of Interventions*. They included chemotherapy protocols of experimental and control groups that were the same or comparable; CHM was used in experimental groups as adjunctive therapy to chemotherapy; placebo or blank controls were eligible; and other treatment was identical in both groups. (4)* Type of Outcome*. The following primary outcomes were included: survival; immediate tumor response, defined as CR or partial response (PR) using the World Health Organization scale; control of nausea and vomiting; and improvement in myelosuppression. The following secondary outcomes were included: performance status evaluated using the Karnofsky performance score (KPS); immune system response, including percent change in T lymphocytes and natural killer (NK) cell activity; QoL; and other side effects, such as alopecia, chemotherapy-related cardiotoxicity, and cognitive dysfunction.

### 2.3. Exclusion Criteria

The exclusion criteria were studies of breast cancer patients with other primary cancer; sample size < 30; CHM used in both groups other than experimental medicine and placebo; studies with > 20% withdrawal and/or dropout rates; nonoriginal studies; or duplicate studies.

### 2.4. Study Selection

Two authors independently screened the trials by first scanning abstracts, titles, and key words to select potential studies based on the inclusion and exclusion criteria. Full articles of the potential studies were obtained for a final determination. If a disagreement occurred, the two authors reviewed the study again, and a third reviewer resolved any disagreement.

### 2.5. Quality Assessment

Methodological quality was assessed using the 5-point Jadad scale [20]. The Jadad scale includes three domains: randomization, blinding, and withdrawals and dropouts. Only studies with a Jadad score of 3, 4, or 5 were included.

### 2.6. Assessment of the Risk of Bias

The risk of bias was assessed using the method recommended by the* Cochrane Handbook* [21]. This tool is a domain-based evaluation in which critical assessments are made separately for the following concepts: randomization, blinding, outcome reporting, and other issues.

### 2.7. Data Management

Two authors independently extracted the following variables: article title; author(s); journal title; year of publication; study design; sample size; sampling and diagnostic procedures; loss to follow-up; exclusions and reasons; baseline characteristics of patients (e.g., age, breast cancer stage); intervention characteristics (e.g., chemotherapy drugs, CHM patterns, CHM type, duration, and dosage); outcome(s); and conclusions. A third reviewer reviewed the extracted data and stored the original data in a secure computer to avoid changes.

### 2.8. Statistical Analysis

We performed a meta-analysis using RevMan 5.2 only when sufficient and suitable data were obtained. We conducted a narrative synthesis when there were too few clinically homogeneous studies for a meta-analysis. We calculated the RR and MD separately for dichotomous and continuous variables. A random effects model was used for pooling because of the clinical heterogeneity of CHM, which includes the complexity of ingredients and different therapeutic methods. We analyzed the pre- and posttreatment data separately to avoid biases from estimations of the values of change from baseline. We assessed heterogeneity using the *χ* ^2^ test, with a *p* value < 0.10 indicating a significant difference. Subgroup analyses were conducted to investigate the source of heterogeneity. Funnel plot asymmetry was used to investigate publication bias when there were at least 10 studies. The total effect was tested using the *Z* test, and *p* < 0.05 was used to identify significant effects.

## 3. Results

### 3.1. Characteristics of Included Studies

**Figure 1:**
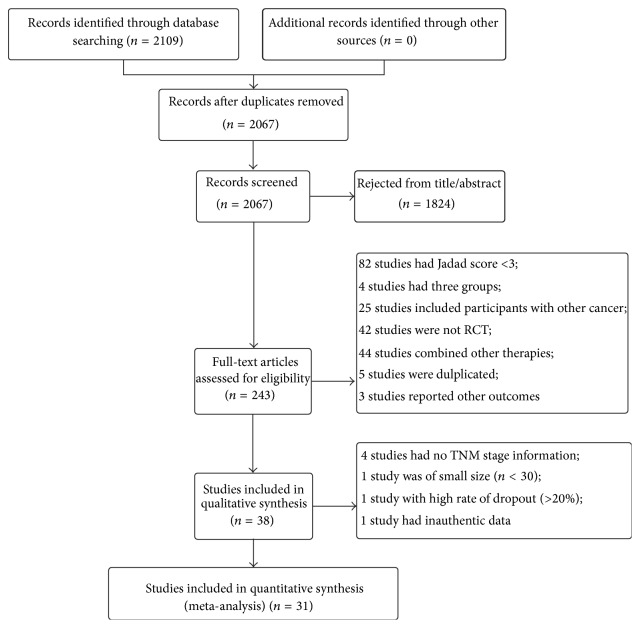
Study flow diagram of the selection process.

The initial search identified 2,109 studies published before October 2014 (Figure 1). Review of the titles and abstracts resulted in the inclusion of 243 studies. Of these, 211 studies were excluded after review of the full text because the studies were duplicates and animal experiments, had a small sample size, had a Jadad score < 3, or did not report the clinical stage. Notably, 90 Chinese studies were excluded because of a low Jadad score, which suggests that Chinese studies in this field are of poor quality. A total of 31 studies were finally included in the meta-analysis, of which 28 were retrieved from Chinese databases and 3 from English databases.

Of the total 2,805 patients who were enrolled in these studies, 1,391 received CTC therapy and 1,319 received chemotherapy alone. Ninety-five patients withdrew or dropped out (Table 1).

**Figure 2:**
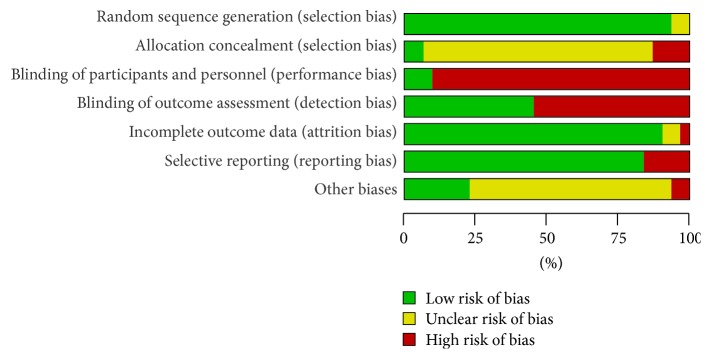
Risk of bias in included studies.

**Figure 3:**
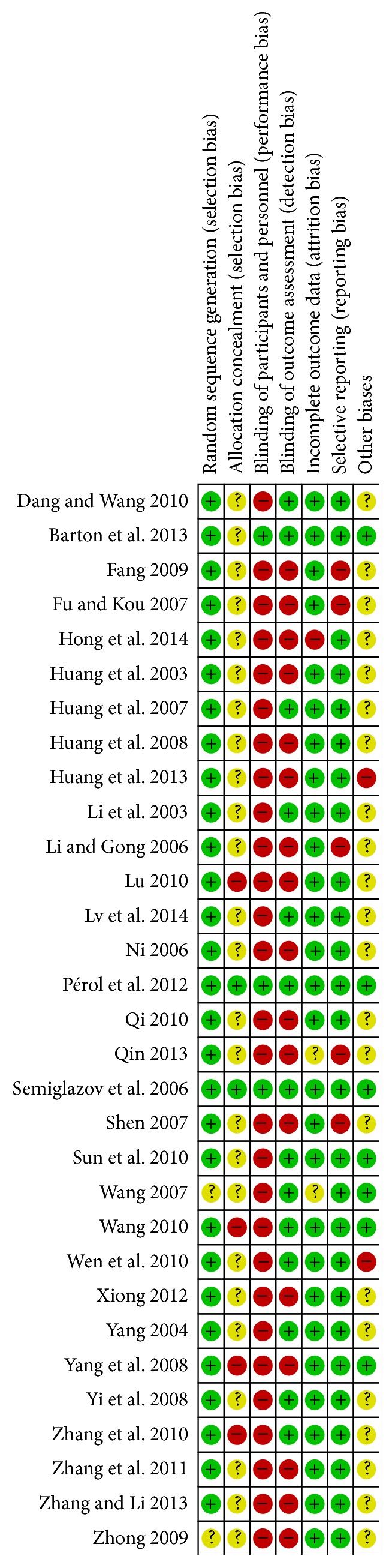
Summary of the risk of bias in included studies.

All trials were described as randomized and had two parallel groups. Concealment of allocation was not reported in any of the Chinese studies. The blinding process was described in the 3 English studies and in only 1 Chinese study. Eight studies reported withdrawals and dropouts. The risk of bias, as assessed using the tool from the Cochrane Collaboration, is shown in Figures 2 and 3.

All recruited patients in the 31 studies were diagnosed using pathological sections, and all studies had a Jadad score of 3, 4, or 5, indicating that the baseline characteristics were comparable among studies (Table 1).

### 3.2. Survival

One study reported 2-year survival with better survival in the CTC group than in the chemotherapy group, but the difference was not significant (RR = 1.15, 95% CI = 0.86–1.53) [26]. However, the median survival time differed significantly between these groups in another study (WMD = 1.90, 95% CI = 0.77–3.03) [25]. However, neither trial was of high quality.

### 3.3. Immediate Tumor Response

**Figure 4:**
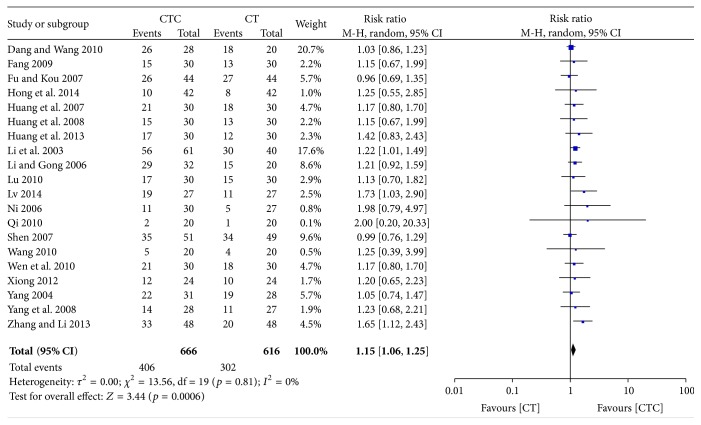
Immediate tumor response in breast cancer (CR + PR).

**Figure 5:**
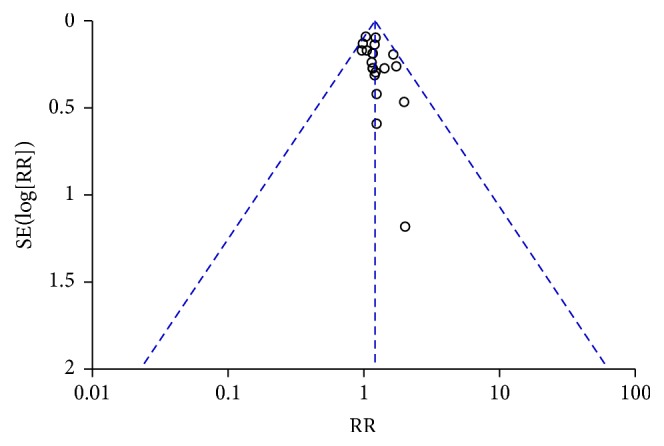
Funnel plot of immediate tumor response in breast cancer (CR + PR).

The immediate tumor response was investigated in 20 studies (1,282 patients). CTC therapy was associated with a significantly higher rate of CR or PR (RR = 1.15, 95% CI = 1.06–1.25, and *p* = 0.0006) (Figure 4) [22, 24–26, 28–35, 37, 40, 43–47, 51]. The exclusion of any one study did not influence the estimated treatment effect. The funnel plot indicated publication bias (Figure 5).

### 3.4. Decrease in Chemotherapy Toxicity

**Figure 6:**
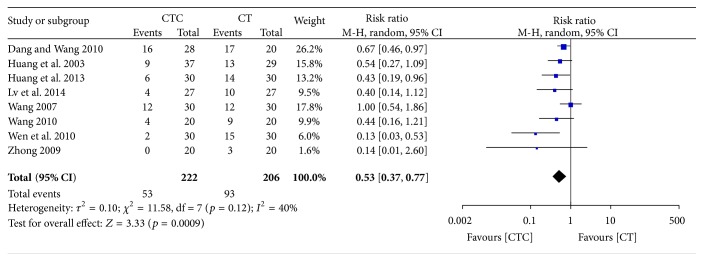
Nausea and vomiting during treatment for breast cancer (toxicity grades II–IV).

**Figure 7:**
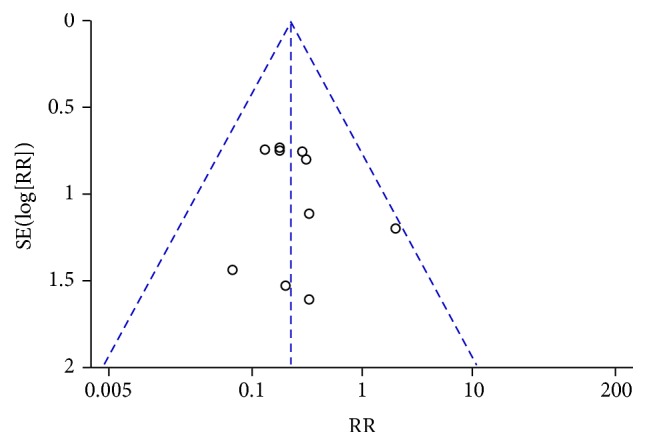
Funnel plot of nausea and vomiting during treatment for breast cancer (toxicity grades III-IV).

**Figure 8:**
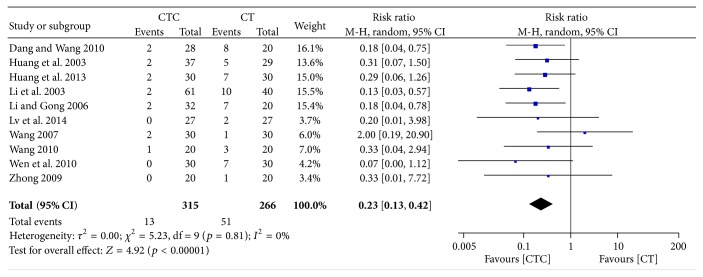
Nausea and vomiting during treatment for breast cancer (toxicity grades III-IV).

Chemotherapy-induced nausea and vomiting (CINV) is one of the most serious and unwanted side effects of chemotherapy [53–55]. The frequency of grade II–IV CINV was significantly lower in the CTC group than in the chemotherapy group (RR = 0.53, 95% CI = 0.37–0.77, and *p* = 0.0009, 8 studies, 428 patients) (Figure 6) [22, 27, 30, 34, 42–44, 52]. The frequency of grade III-IV CINV was significantly lower in patients undergoing CTC therapy (RR = 0.23, 95% CI = 0.13–0.42, and *p* < 0.00001, 10 studies, 581 patients) (Figures 7 and 8) [22, 27, 30–32, 34, 42–44, 52]. However, one study, conducted in France, reported no differences in scores for nausea, vomiting, or global emesis on the Functional Living Index-Emesis tool between the two arms (WMD = 0.02, 95% CI = −0.29–0.33) [36], and another study reported a significant difference [41]. These two studies were not pooled for analysis because of the different data types.

**Figure 9:**
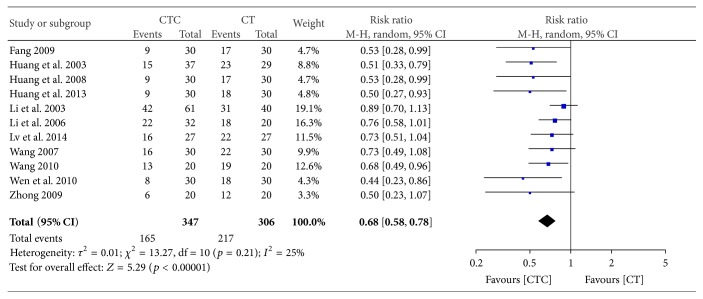
Reductions in WBCs during breast cancer treatment (toxicity grades I–IV).

**Figure 10:**
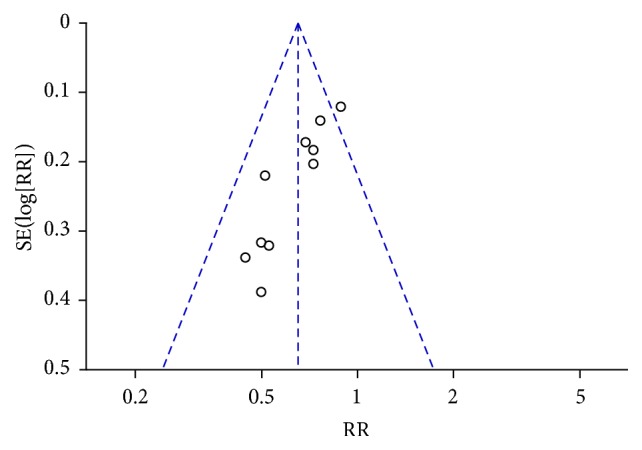
Funnel plot of the reduction in WBCs during breast cancer treatment (toxicity grades I–IV).

**Figure 11:**
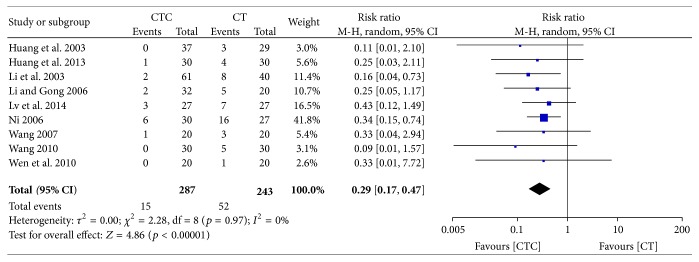
Reduction in WBCs during breast cancer treatment (toxicity grades III-IV).

Grade III-IV-induced reductions in white blood cell (WBC) counts were significantly less frequent in the CTC group (RR = 0.68, 95% CI = 0.58–0.78, and *p* < 0.00001, 11 studies, 653 patients) (Figures 9 and 10) [24, 27, 29–32, 34, 42–44, 52]. Grade III-IV-induced reductions in WBC counts were also significantly less frequent in the CTC group (RR = 0.29, 95% CI = 0.17–0.47, and *p* < 0.00001, 9 studies, 530 patients) (Figure 11) [27, 30–32, 34, 42–44, 52].

**Figure 12:**
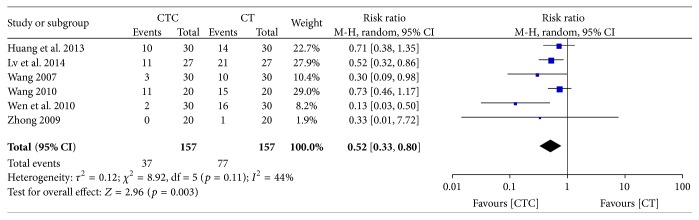
Reduction in platelets during breast cancer treatment (toxicity grades I–IV).

Figure 12 shows that grade I–IV-induced reductions in platelets decreased in the CTC group (RR = 0.52, 95% CI = 0.33–0.80, and *p* = 0.003, 6 studies, 314 patients) [30, 34, 42–44, 52].

**Figure 13:**
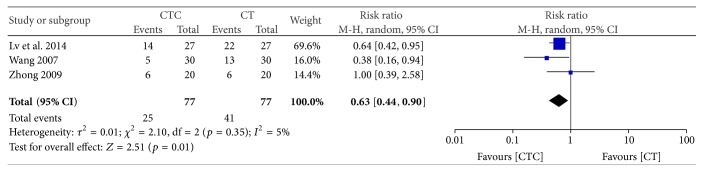
Reduction in hemoglobin during breast cancer treatment (toxicity grades I–IV).

Grade I–IV-induced reductions in hemoglobin were significantly less frequent in participants undergoing combined treatment (RR = 0.63, 95% CI = 0.44–0.90, and *p* = 0.001, 3 studies, 154 patients) (Figure 13) [34, 42, 52].

### 3.5. Performance Status

**Figure 14:**
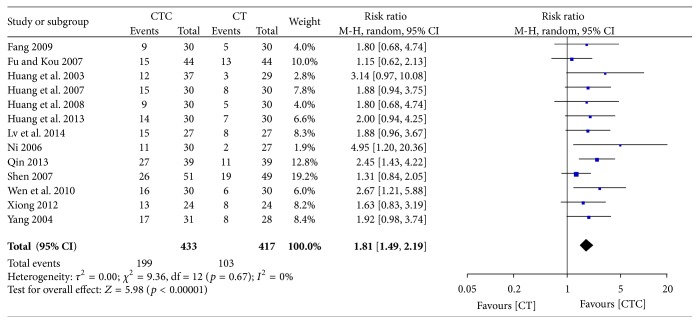
Improvement in KPS during breast cancer treatment.

**Figure 15:**
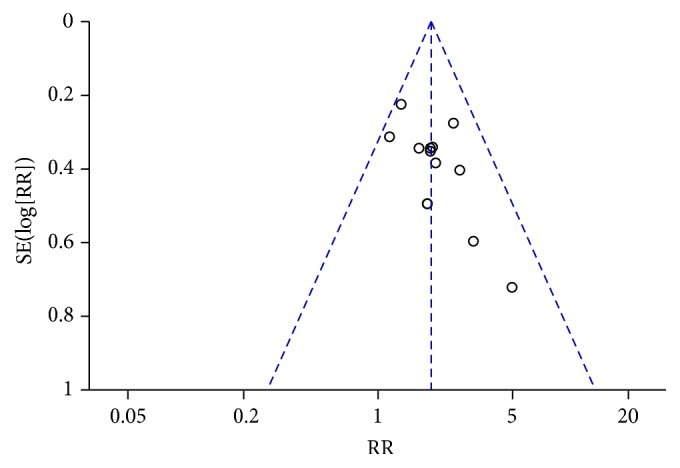
Funnel plot of improvement in KPS during breast cancer treatment.

**Figure 16:**
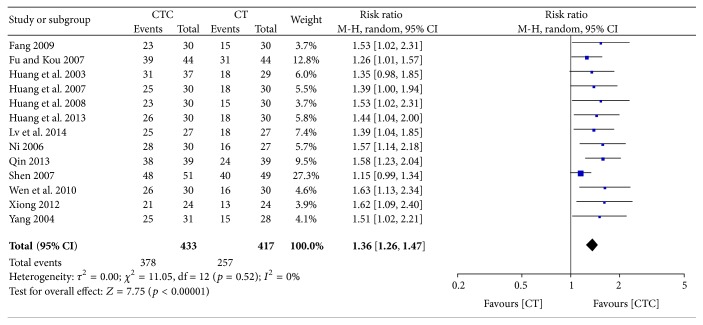
Improvement and stabilization of performance status during breast cancer treatment.

**Figure 17:**
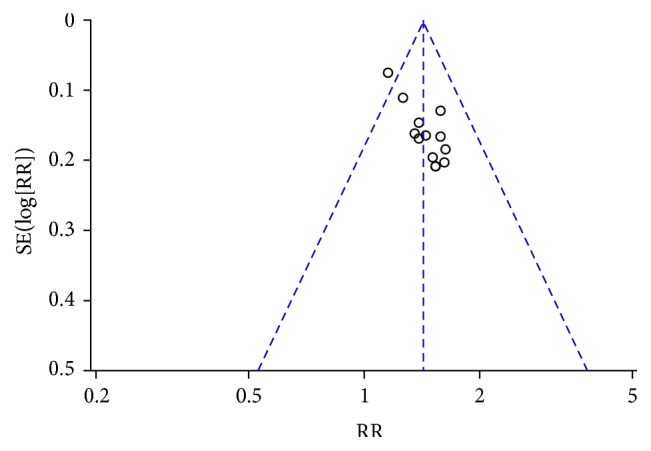
Funnel plot of improvement and stabilization of performance status during breast cancer treatment.

Different types of KPS data were calculated in the studies. The first type was the improvement or stabilization of the KPS using a cutoff value of a 10-point change. The second type was the pre- and posttreatment KPS values. KPS improvement (≥10-point increase) was significantly better in the CTC group (RR = 1.81, 95% CI = 1.49–2.19, and *p* < 0.00001, 13 studies, 850 patients) (Figures 14 and 15) [24, 25, 27–30, 34, 35, 38, 40, 44–46]. The rate of improvement and stabilization (change in KPS of >0) was also significantly higher in participants receiving CTC (RR = 1.36, 95% CI = 1.26–1.47, and *p* < 0.00001, 13 studies, 850 patients) (Figures 16 and 17) [24, 25, 27–30, 34, 35, 38, 40, 44–46].

**Figure 18:**
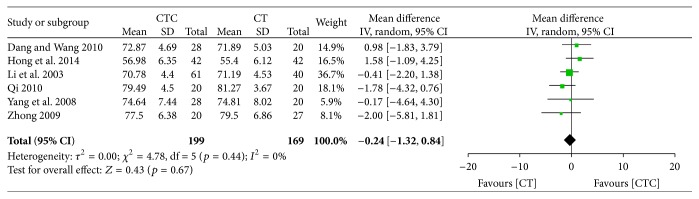
KPS before breast cancer treatment.

**Figure 19:**
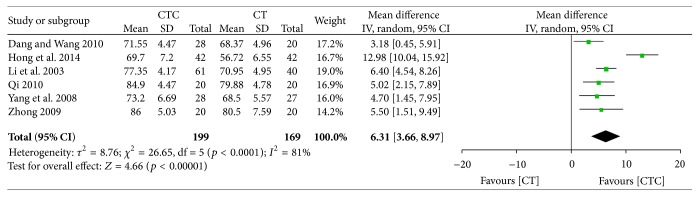
KPS after breast cancer treatment.

Pre- and posttreatment KPSs were reported in 6 studies. The difference in pretreatment KPS was not significant between the two arms (MD = −0.24, 95% CI = −1.32–0.84, and *p* = 0.67, 6 studies, 368 patients) (Figure 18) [22, 26, 31, 37, 47, 52]. The posttreatment KPS was significantly higher in the CTC group than in the chemotherapy group (MD = 6.31, 95% CI = 3.66–8.97, and *p* < 0.00001, 6 studies, 368 patients) (Figure 19).

### 3.6. Immunostimulation

**Figure 20:**
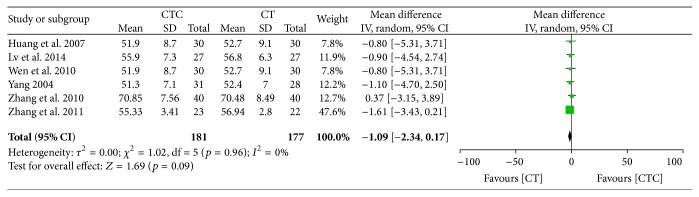
CD3^+^ before treatment.

**Figure 21 fig21:**
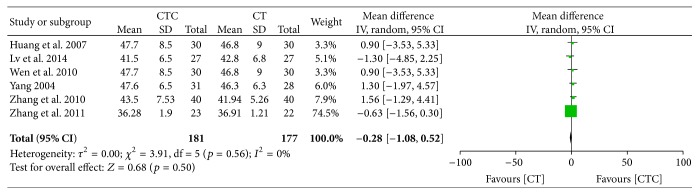
CD4^+^ before treatment.

**Figure 22 fig22:**
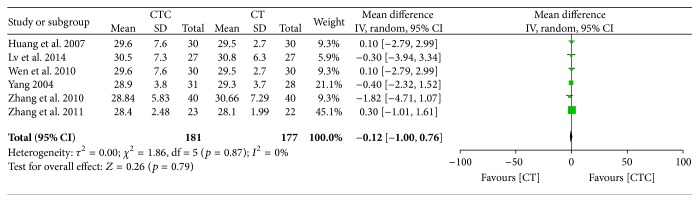
CD8^+^ before treatment.

**Figure 23 fig23:**
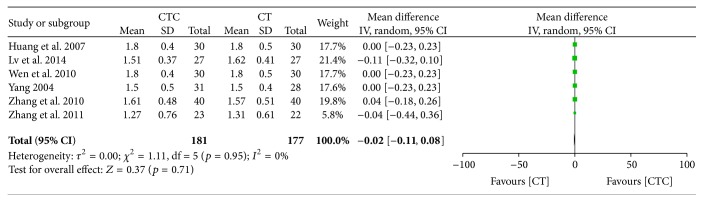
CD4^+^/CD8^+^ before treatment.

**Figure 24:**
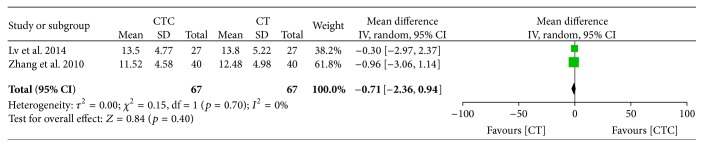
Natural killer cell level before treatment.

Figures 20 [Fig fig21] [Fig fig22] [Fig fig23]–24 show that, in the pooled studies, the differences in pretreatment T lymphocytes (CD3^+^, CD4^+^, CD8^+^, and CD4^+^/CD8^+^) and NK cell levels between the two arms were not significant (CD3^+^, MD = −1.09, 95% CI = −2.34–0.17, *p* = 0.09, and *I* ^2^ = 0%; CD4^+^, MD = −0.28, 95% CI = −1.08–0.52, *p* = 0.50, and *I* ^2^ = 0%; CD8^+^, MD = −0.12, 95% CI = −0.10–0.76, *p* = 0.79, and *I* ^2^ = 0%; CD4^+^/CD8^+^, MD = −0.02, 95% CI = −0.11–0.08, *p* = 0.71, and *I* ^2^ = 0%; NK cells, MD = −0.71, 95% CI = −2.36–0.94, *p* = 0.40, and *I* ^2^ = 0%).

**Figure 25:**
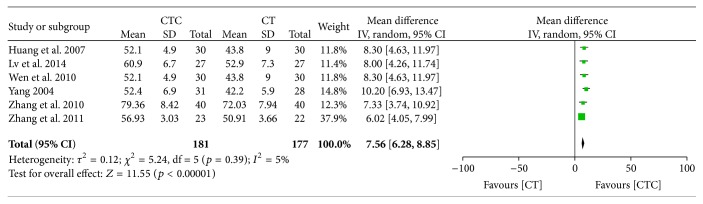
CD3^+^ after breast cancer treatment.

**Figure 26:**
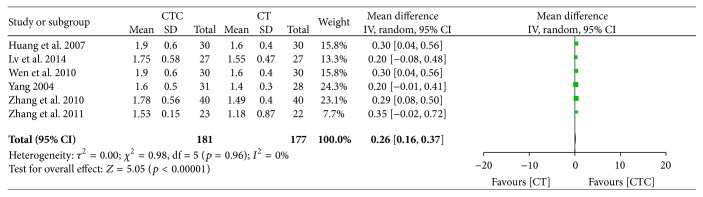
CD4^+^/CD8^+^ after breast cancer treatment.

**Figure 27:**
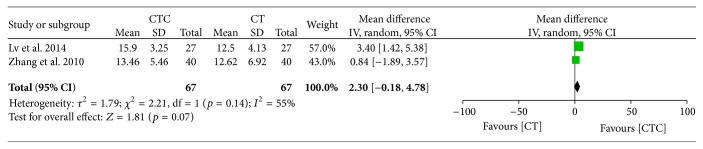
Natural killer cell level after breast cancer treatment.

CTC therapy showed an advantage for CD3^+^ and CD4^+^/CD8^+^ cells posttreatment (MD = 7.56, 95% CI = 6.28–8.85, *p* < 0.00001, and *I* ^2^ = 5%, 6 studies, 358 patients, and MD = 0.26, 95% CI = 0.16–0.37, and *p* < 0.00001, 6 studies, 358 patients, resp.) (Figures 25 and 26) [28, 34, 44, 46, 49, 50]. However, the result indicates that the posttreatment NK cell level was not significantly different between the CTC and chemotherapy groups (MD = 2.30, 95% CI = −0.18–4.78, and *p* = 0.07, 2 studies, 134 patients) (Figure 27) [34, 49]. Heterogeneity existed among the included studies in terms of CD4^+^ and CD8^+^ cells posttreatment.

**Figure 28:**
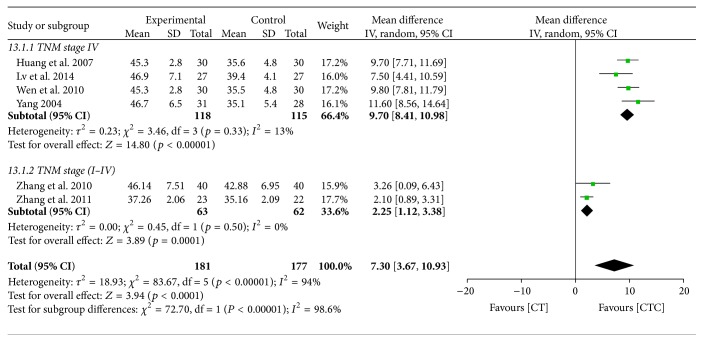
CD4^+^ after breast cancer treatment.

**Figure 29:**
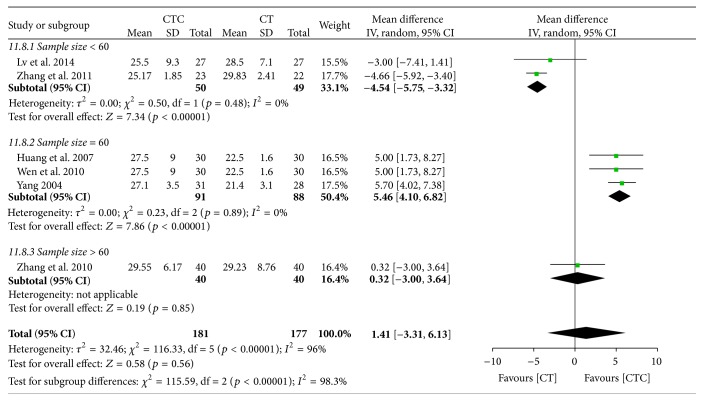
CD8^+^/CD8^+^ after breast cancer treatment.

CTC therapy also resulted in significantly better posttreatment CD4^+^ levels (MD = 7.30, 95% CI = 3.67–10.93, *p* < 0.0001, and *I* ^2^ = 94%, 6 studies, 358 patients) (Figure 28). A subgroup analysis indicated that TNM stage may have affected the homogeneity. In the pooled studies, there were no significant differences in posttreatment CD8^+^ levels between the two arms (MD = 1.41, 95% CI = −3.31–6.13, *p* = 0.56, and *I* ^2^ = 96%, 6 studies, 356 patients) (Figure 29). The differences in sample size may have contributed to the heterogeneity of the studies.

### 3.7. QoL

Six studies reported on QoL, but we were not able to pool these results because each study used a different scale and data type. The study by Semiglazov et al. was of high quality and low risk, and the QoL was assessed using 3 FACT-G subscales (physical, emotional, and functional well-being) [39]. The intervention group exhibited improvements in the FACT-G total score and in the physical, emotional, and functional well-being scores, and the placebo group had poorer scores. Four studies that reported significantly improved QoL using combined therapy, as assessed using the Chinese version of the FACT-G, were of mixed quality [37, 42, 50, 52]. Two studies reported QoL as assessed using the Chinese version of the EORTC QLQ C-30. Zhong observed improvements in the physical and emotional subscales and the overall health QoL score with CTC therapy [52]. Wang reported significant within-group improvements in some subscales in both groups, but there were no details of comparisons between groups [43].

### 3.8. Other Outcomes

**Figure 30:**
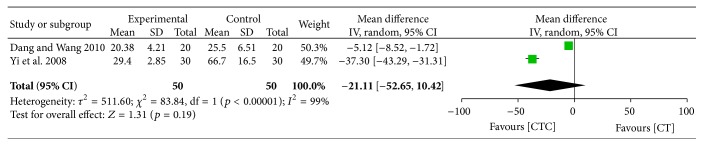
CK-MB (U/L) after breast cancer treatment.

One high-quality study evaluated CTC therapy for the prevention of chemotherapy-related cognitive dysfunction and reported no significant difference between the two arms [23]. Two studies that reported chemotherapy-related cardiotoxicity had significant heterogeneity, and CTC did not have a significant advantage in the prevention of creatinine kinase-MB isoenzyme (WMD = −21.11, 95% CI = −52.65–10.42, and *p* = 0.19) (Figure 30) [22, 48].

## 4. Discussion

Treatment of breast cancer using CHM has been described in Chinese medical texts for more than 2,000 years. Accepting TCM as science rather than myth remains a challenge in Western countries despite the recent increase in the use of CHM.

This review has several limitations. First, we did not identify studies in languages other than Chinese and English. CAM use is reportedly high in East Asia, where CHM originated, with use rates of 29–83% in South Korea and 50% in one study in Japan [56, 57]. Therefore, additional studies should be identified from or conducted in these areas to further investigate the efficacy of CHM. Second, all of the included Chinese studies had relatively small sample sizes, ranging from 40 to 101 participants. None of these studies reported the details of sample size calculation. Third, the Chinese trials did not clearly report allocation concealment or blinding, and none of the Chinese studies were placebo controlled or double blinded, which could have resulted in bias and an overestimation of CTC efficacy [58]. Publication bias may also have existed. The asymmetry of the funnel plot may be the result of an insufficient number of trials and significant statistical heterogeneity (Figures 5, 7, 15, and 17). There were also different data types and assessment methods for outcomes, which may have resulted in statistical heterogeneity.

We also cannot ignore the low quality of the included trials; however, that may not be a sound reason to exclude a systematic review. A systematic review embraces the features of systematization and comprehensiveness, which differentiate it from a normal review. In addition, the CHM used differed significantly among trials. Inevitably, the pharmacological actions of these treatments would not be the same. A random effects model was used for pooling because of the clinical heterogeneity. Because of this limitation, we cannot draw a convincing conclusion. Nevertheless, the problems with the current studies identified in this review are significant, and a great deal of work needs to be done to evaluate the efficacy of CAM using a modern and rigorous methodology.

Only 3 studies evaluated the Zheng TCM pattern, which is another key limitation of the included studies [37, 42, 52]. Zheng, also known as syndrome or pattern, is the core concept in TCM, and it describes the entire physiological and/or pathological pattern of the patient [59]. Zheng is usually evaluated through a comprehensive analysis of clinical signs and symptoms. TCM practitioners collect the signs by inspection, auscultation, olfaction, inquiry, pulse, and palpation. TCM practitioners in clinical practice prescribe CHM based on Zheng.

CHM therapy is more efficacious when based on the correct judgment of the Zheng classification according to the Chinese medical system. One clinical study found that the therapeutic effect of CHM for the treatment of irritable bowel syndrome was more sustainable when based on the TCM pattern than on standard treatment [60]. The key role of Zheng in TCM should not be ignored despite the controversial results reported by other clinical studies, which indicate that the efficacy of Zheng-based treatment is not advantageous over standard treatment [61–63]. Patients are not administered the same CHM for a long period of time in real practice, and the treatments reported in clinical trials did not follow a pattern that is commonly used in actual clinical practice because Zheng is dynamic during the treatment course. The biggest challenge in the exploration of Zheng-based CHM therapy using an RCT is the standardization of Zheng. Currently, the process of Zheng is highly subjective, and a nationwide and objective process is needed to improve its use. Randomized, multicenter trials should be conducted for this purpose. Analyses of Zheng at the molecular level may also enable acceptance of TCM on a scientific basis for the West.

Table 2 lists the herbal medicines that were commonly used for the treatment of breast cancer in the identified studies in this review. The pooled data in this review demonstrated that the adjunctive use of CHM with chemotherapy may improve immediate tumor response and performance status and reduce the occurrence of adverse events associated with chemotherapy. We were unable to verify whether CHM helped stimulate the immune system, as measured using CD3^+^, CD4^+^, CD8^+^, and CD4^+^/CD8^+^ cells, because of the mixed quality and significance of the included studies. The evidence is too limited to make any confident conclusions. These results suggest that combined therapy has potential benefits for breast cancer patients. The finding of CHM efficacy as an adjunctive therapy for breast cancer is similar to the findings of other reviews for hepatocellular carcinoma, non-small-cell lung cancer, colorectal cancer, and nasopharyngeal carcinoma [58, 64–66]. A recent systematic review involving 8 RCTs showed that CHM combined with conventional therapy for breast cancer was efficacious in improving QoL and decreasing hot flashes, but this study did not identify as many clinical trials as it could have [67]. Breast cancer patients undergoing chemotherapy and/or endocrine therapy were included in that review, and the effect of CHM for breast cancer should have been examined separately in those two groups. In addition, the review focused on the effects on QoL and hot flashes but did not evaluate other cancer-related symptoms. Finally, the reviewers only presented a narrative synthesis without a meta-analysis, which made the conclusion unconvincing.

GRADE should be applied to judge the evidence and make recommendations regarding the application of CHM in the treatment of breast cancer. The present study suggests that recommendations for CHM combined with chemotherapy could be made for breast cancer, but TCM may be too complex to be immediately adopted by physicians in Western countries. The most fundamental and often-overlooked challenge is the lack of a 1 : 1 correlation between modern allopathic and Chinese holistic medical approaches [68]. We cannot make specific recommendations despite the rapid increase in the use of CHM and reported potential benefits because of the complexity of this system and the variable data.

Current evidence on the use of CHM as an adjunctive treatment with chemotherapy for breast cancer remains equivocal. Our findings highlight the poor quality of Chinese studies, and additional well-designed RCTs addressing the role of CHM are warranted. The lack of molecular-based evidence for CHM and Zheng has resulted in a limited understanding and acceptance of CHM and TCM in Western countries. We believe that researchers should immediately explore a CHM-based cure, and CHM should be applied to routine care as soon as conclusive data are available [69]. For researchers devoted to the promotion of TCM or CHM, numerous barriers need to be addressed, including the standardizations of the Zheng classification and herbal agents, appropriate study designs, and the identification of the mechanisms of CHM at the molecular level.

## Figures and Tables

**Table 1:** Characteristics of the included studies for the use of CHM as an adjunct for chemotherapy in breast cancer patients.

Study	Number of participants/dropouts	TNM stage	Duration (weeks)	Control group interventions	CHM interventions	Outcome(s)	Jadad scale score
Dang and Wang 2010 [22]	48/0	I–III	9	CTF	Aidi injection	Tumor response, cardiotoxicity, KPS, and chemotoxicity	3
Barton et al. 2013 [23]	210/44	I–IV	52	TE/TEC	Ginkgo Biloba	Cognitive dysfunction	5
Fang 2009 [24]	60/0	III/IV	3	CTF	Shenqi Wuweizi pill	Tumor response, KPS, and chemotoxicity	3
Fu and Kou 2007 [25]	88/0	IV	8	NP	Aidi injection	Tumor response, KPS, chemotoxicity, and median survival	3
Hong et al. 2014 [26]	91/7	II–IV	18	TEC	Xihuang pill	Tumor response, KPS, and overall survival	3
Huang et al. 2003 [27]	66/0	II–IV	4	CMF	Bazhen decoction	KPS and chemotoxicity	3
Huang et al. 2007 [28]	60/0	IV	6	CTF	Jianpi Xiaoji decoction	Tumor response, KPS, and immune system	3
Huang et al. 2008 [29]	60/0	III/IV	6	CTF	Shenqi Fuzheng injection	Tumor response, KPS, and chemotoxicity	3
Huang et al. 2013 [30]	60/0	IV	18–24	CEF	Huangqi injection	Tumor response, KPS, chemotoxicity, and median survival	3
Li et al. 2003 [31]	101/0	I–III	6~9	CMF	Rukang I prescription	Tumor response, KPS, and chemotoxicity	3
Li and Gong 2006 [32]	52/0	I–III	9	CEF	Aidi injection	Tumor response and chemotoxicity	3
Lu 2010 [33]	60/0	III/IV	6	CAF	Huangqi injection	Tumor response	3
Lv et al. 2014 [34]	54/0	IV	12–16	FAC	Yiqi Huoxue Huayu decoction	Tumor response, KPS, chemotoxicity, and immune system	3
Ni 2006 [35]	57/0	IV	13	Docetaxel + THP	Gaolisheng injection	Tumor response and chemotoxicity	3
Pérol et al. 2012 [36]	430/27	I–IV	8–12	FAC	Cocculine	Chemotherapy-induced emesis	5
Qi 2010 [37]	40/0	II–IV	6	TE	Yiqi Yangxue Shugan decoction	Tumor response, KPS, quality of life, and chemotoxicity	3
Qin 2013 [38]	78/0	I–IV	21	CAF	Fuzheng Qiqi Jiedu decoction	KPS	3
Semiglazov et al. 2006 [39]	352/21	I–III	16–24	CMF	Mistletoe extract	Quality of life	5
Shen 2007 [40]	100/0	IV	6	NVB + THP	Aiyishu injection	Tumor response and KPS	3
Sun et al. 2010 [41]	86/0	II–IV	3	CAF/AC	Zaofan pill	Chemotoxicity	3
Wang 2007 [42]	60/0	II-III	9	CEF	Taohong Siwu decoction	Quality of life and chemotoxicity	3
Wang 2010 [43]	40/0	II–IV	8	TE	Yiqijianpi Huayujiedu decoction	Tumor response, quality of life, and chemotoxicity	3
Wen et al. 2010 [44]	60/0	IV	3	TA	Fuzheng Xiaoyan prescription	Tumor response, quality of life, chemotoxicity, and immune system	3
Xiong 2012 [45]	48/0	IV	6	NVB + CAP	Fuzhengxiaoji decoction	Tumor response and KPS	3
Yang 2004 [46]	59/0	IV	6	NVB + THP	Aidi injection	Tumor response, KPS, and immune system	3
Yang et al. 2008 [47]	59/4	III B-IV	6–8	NP	Guben Yiliu II decoction	Tumor response and KPS	3
Yi et al. 2008 [48]	60/0	IV	12	DOX	Ginkgo Biloba	Cardiotoxicity	3
Zhang et al. 2010 [49]	80/0	I/II/III	18	CEF	Huangqi Taohong decoction	Immune system	3
Zhang et al. 2011 [50]	45/0	III-IV	6	CTF	Fuzheng Quyu Jiedu prescription	Immune system and quality of life	3
Zhang and Li 2013 [51]	96/0	II-III	3-4	CTF	Tiaogan Jianpi prescription	Tumor response	3
Zhong 2009 [52]	40/0	I–IV	6	TE	Shugan Tiaoli Chongren decoction	Quality of life, KPS, and chemotoxicity	3

KPS: Karnofsky performance score.

**Table 2:** Herbal medicines commonly used in the treatment of breast cancer.

Chinese herbal medicine	Frequency	
	Count	%
Radix Astragalus	20	9.22
Rhizoma Atractylodis Macrocephalae	12	5.53
Poria	10	4.61
Angelica	8	3.69
*Codonopsis pilosula*	8	3.69
Radix Glycyrrhizae	7	3.23
*Ligustrum lucidum*	7	3.23
*Oldenlandia diffusa*	6	2.76
Pericarpium Citri Reticulatae	6	2.76
Panax	6	2.76
Pseudobulbus Cremastrae seu Pleiones	6	2.76
